# Supplementary material for: Band Phosphorus and Sulfur Fertilization as Drivers of Efficient Management of Nitrogen of Maize (Zea mays L.)
Source: Plants (Basel). 2022 Jun 23;11(13):1660. doi: 10.3390/plants11131660 (PMC9268923; doi:10.3390/plants11131660)
Supplement: Supplementary file 1 [file plants-11-01660-s001.zip › plants-1757725-supplementary.pdf]

# Supplementary material

## Band phosphorus and sulfur fertilization as drivers of efficient management of nitrogen of maize (*Zea mays* L.)

Przemysław Barłóg[1*], Remigiusz Łukowiak[1] and Lukáš Hlisnikovský[2]

Department of Agricultural Chemistry and Environmental Biogeochemistry, Poznan University of Life Sciences, Wojska Polskiego 71F, 60-625 Poznan, Poland; remigiusz.lukowiak@up.poznan.pl (R.Ł.)

[2] Department of Nutrition Management, Crop Research Institute, Drnovská 507, CZ-161 01 Prague 6, Ruzyně, the Czech Republic; l.hlisnik@vurv.cz (L.H.)

* Correspondence: przemyslaw.barlog@up.poznan.pl; Tel.: +48-618-48-77-88 (P.B.)

**Treatments of NP(S) fertilization were as follows:** $P_0$ – control; $P_1$ – 8.7; $P_2$ – 17.4 and $P_3$ – 26.2 kg ha$^{-1}$ of P (17%; 33% and 67% of total P uptake for grain yield = 9.0 t ha$^{-1}$). The N rates were as follows: $N_0$ - control; $N_1$ – 60; $N_2$ – 120 and $N_3$ –180 kg ha$^{-1}$ of N. A nitrogen rate 180 kg N ha-1 corresponded to 100% of maize requirement for this nutrient in mineral fertilizers. The effect of mineral fertilization with NPS was compared to the ab-solute control (AC), without fertilization with these components. NPS were applied to the soil using fertilizer NPS(+Zn) in the proportion 20-20-35+0.3 (calculated for N, $P_2O_5$, $SO_3$, Zn). The fertilizer is based on two main chemical compounds: di-ammonium phosphate, DAP [$(NH_4)_2HPO_4$] and ammonium sulfate [$(NH_4)_2SO_4$]. Therefore, on plots with localized fertilization P1, P2 and P3, the doses of ammonium nitrogen ($NH_4$-N) were 20; 40 and 60 kg ha$^{-}$1 of N, and those of sulfur were 14, 28 and 42 kg ha$^{-1}$ of S, respectively. In order to simplify the notation of fertilization treatments, the NPS rates were recorded as P rates. The NPS fertilizer was applied while sowing the seeds. The fertilizer was banded to 5 cm below and away from the seeds. In treatments without NPS fertilization or with low total N rates, maize requirement for N was supplemented with ammonium nitrate (34% N) – broadcast application immediately before sowing. Potassium fertilization was carried out in early spring in the form of potassium salt K(S,Mg) in proportion 41(15,6.5) at the rate of 66.4 kg ha$^{-1}$ of K, regardless of the NPS treatment.

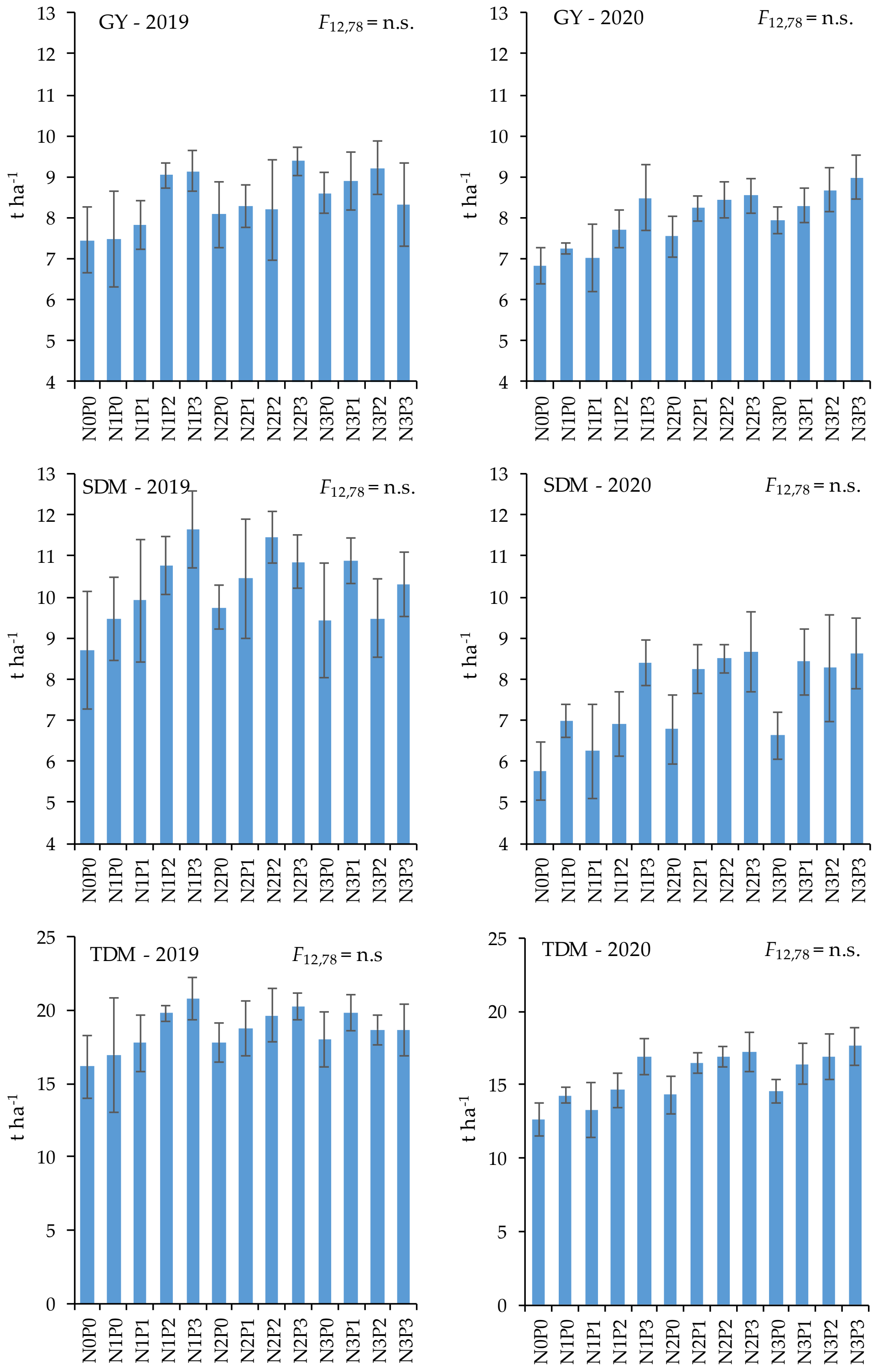


**Figure S1.** Maize grain yield (GY), crop residues biomass (SDM) and total dry matter (TDM) depending on the year (2019-2020) and fertilization treatments. Means within a column followed by the same letter indicate a lack of significant difference between the fertilized treatments (HSD test). Hatched bars represent 2×SEM ranges.

**Table S1.** Maize yield components depending on the year and fertilization treatments.

| Year Treatment | Plant density No. ha$^{-1}$ | Cob No. plant$^{-1}$ | Thousand grain weight, g | No. of grains cob$^{-1}$ |
|---|---|---|---|---|
| | | Effect of the year (Y) | | |
| 2019 | 74945 ±728 | 0.99 ±0,01 | 228.8 ±1,9 $^{b}$ | 517.6 ±11,8 $^{a}$ |
| 2020 | 75000 ±750 | 0.99 ±0,01 | 311.6 ±3,7 $^{a}$ | 447.8 ±7,8 $^{b}$ |
| | | Effect of the treatment (T) | | |
| N0P0 | 78214 ±1663 | 0.99 ±0,01 | 245.9 ±11,1 $^{b}$ | 459.2 ±33,1 |
| N1P0 | 74881 ±423 | 0.99 ±0,02 | 266.6 ±18,1 $^{ab}$ | 447.2 ±39,4 |
| N1P1 | 75357 ±674 | 0.98 ±0,01 | 252.0 ±15,6 $^{ab}$ | 478.9 ±16,9 |
| N1P2 | 74167 ±650 | 0.99 ±0,01 | 273.4 ±14,7 $^{ab}$ | 508.0 ±17,7 |
| N1P3 | 74167 ±404 | 1.00 ±0,01 | 273.7 ±17,5 $^{ab}$ | 499.6 ±29,2 |
| N2P0 | 74167 ±826 | 0.98 ±0,01 | 262.7 ±14,3 $^{ab}$ | 497.1 ±25,5 |
| N2P1 | 75833 ±892 | 0.99 ±0,02 | 279.3 ±18,8 $^{ab}$ | 473.0 ±22,3 |
| N2P2 | 76310 ±1034 | 0.98 ±0,01 | 280.2 ±20,2 $^{a}$ | 464.0 ±25,3 |
| N2P3 | 73214 ±1347 | 1.01 ±0,01 | 274.3 ±17,0 $^{ab}$ | 509.3 ±34,6 |
| N3P0 | 74881 ±2888 | 1.00 ±0,01 | 273.3 ±18,8 $^{ab}$ | 474.5 ±37,4 |
| N3P1 | 73929 ±461 | 1.00 ±0,01 | 280.6 ±17,3 $^{a}$ | 479.2 ±30,4 |
| N3P2 | 74643 ±961 | 0.99 ±0,005 | 276.8 ±16,3 $^{ab}$ | 494.2 ±35,8 |
| N3P3 | 74881 ±424 | 0.98 ±0,01 | 274.0 ±20,6 $^{ab}$ | 490.9 ±21,7 |
| | | ANOVA results (significance level) | | |
| Year | n.s. | n.s. | *** | *** |
| Treatment | n.s. | n.s. | * | n.s. |
| Y × T | n.s. | n.s. | n.s. | n.s. |

***, **, * significant at $p < 0.001$; $p < 0.01$; $p < 0.05$, respectively; n.s. – non significant; means within a column followed by the same letter indicate a lack of significant difference between the fertilized treatments (HSD test).

**Table S2.** Dry matter (DM) at two growth sages (7 leaf and start of lowering), nitrogen concentration in DM; accumulation N in particular plant organs and total N accumulation by maize depending on the year and fertilization treatment

| Treatment | Maize DM, g plant$^{-1}$ | | Nitrogen concentration g kg$^{-1}$ DM | | | | Nitrogen accumulation g ha$^{-1}$ | | | | |
|---|---|---|---|---|---|---|---|---|---|---|---|
| | **7 leaf** | **Flowering** | **7 leaf** | **Flowering** | **Grain** | **Crop resi-dues** | **7 leaf** | **Flowe-ring** | **Grain** | **Crop resi-dues** | **Total** |
| | | | | | Year 2019 | | | | | | |
| N0P0 | 7.6 | 81.3 | 37.8 | 20.5 | 14.7 | 10.0 | 22.1 | 125.2 | 109.9 | 87.4 | 197.3 |
| N1P0 | 8.1 | 91.3 | 36.6 | 26.2 | 16.4 | 11.7 | 22.7 | 179.5 | 122.5 | 110.6 | 233.0 |
| N1P1 | 9.5 | 78.9 | 40.2 | 27.7 | 16.9 | 12.4 | 28.6 | 163.9 | 132.1 | 123.4 | 255.5 |
| N1P2 | 9.2 | 93.2 | 41.2 | 26.4 | 17.4 | 9.7 | 28.4 | 184.4 | 157.0 | 104.4 | 261.4 |
| N1P3 | 9.3 | 97.3 | 42.2 | 25.0 | 17.8 | 12.7 | 29.4 | 182.2 | 162.7 | 147.3 | 310.0 |
| N2P0 | 9.8 | 89.4 | 37.3 | 27.6 | 18.0 | 12.8 | 27.8 | 185.2 | 145.8 | 124.6 | 270.4 |
| N2P1 | 10.0 | 89.6 | 40.9 | 27.6 | 17.7 | 11.0 | 30.8 | 185.5 | 146.5 | 115.0 | 261.4 |
| N2P2 | 8.7 | 92.1 | 41.0 | 26.0 | 18.4 | 12.4 | 27.0 | 179.6 | 150.8 | 142.4 | 293.1 |
| N2P3 | 10.2 | 89.6 | 40.8 | 27.1 | 16.8 | 12.6 | 31.3 | 182.3 | 157.4 | 136.8 | 294.2 |
| N3P0 | 10.2 | 93.4 | 39.8 | 26.2 | 18.2 | 12.8 | 31.0 | 183.6 | 156.9 | 120.8 | 277.6 |
| N3P1 | 11.1 | 85.5 | 38.4 | 24.6 | 18.7 | 10.2 | 32.5 | 157.8 | 166.5 | 111.2 | 277.6 |
| N3P2 | 10.5 | 80.8 | 42.0 | 27.3 | 18.0 | 13.4 | 33.1 | 165.7 | 165.9 | 126.6 | 292.5 |
| N3P3 | 10.2 | 95.0 | 41.9 | 27.3 | 18.4 | 12.8 | 32.3 | 194.4 | 152.7 | 131.8 | 284.5 |
| | | | | | Year 2020 | | | | | | |
| N0P0 | 5.7 | 83.5 | 35.0 | 15.4 | 13.8 | 10.5 | 14.9 | 96.5 | 94.3 | 60.4 | 154.7 |
| N1P0 | 6.8 | 99.6 | 39.1 | 15.4 | 14.7 | 10.5 | 19.9 | 115.0 | 106.6 | 73.6 | 180.3 |
| N1P1 | 6.2 | 118.7 | 43.0 | 17.3 | 15.9 | 10.9 | 20.0 | 154.1 | 112.0 | 67.7 | 179.7 |
| N1P2 | 7.8 | 102.7 | 42.1 | 19.3 | 16.3 | 11.0 | 24.5 | 148.8 | 125.7 | 76.1 | 201.8 |
| N1P3 | 8.1 | 127.3 | 42.1 | 17.3 | 17.0 | 11.3 | 25.7 | 165.2 | 144.1 | 95.3 | 239.4 |
| N2P0 | 6.4 | 102.2 | 44.5 | 18.0 | 15.4 | 12.4 | 21.5 | 138.2 | 116.1 | 84.0 | 200.1 |
| N2P1 | 7.0 | 121.8 | 40.3 | 19.4 | 16.3 | 10.1 | 21.2 | 177.1 | 134.1 | 83.0 | 217.1 |
| N2P2 | 9.0 | 123.6 | 43.5 | 19.8 | 17.0 | 11.2 | 29.4 | 183.3 | 143.0 | 95.2 | 238.2 |
| N2P3 | 7.7 | 121.3 | 41.2 | 16.5 | 16.6 | 11.9 | 23.9 | 149.6 | 142.1 | 102.9 | 245.0 |
| N3P0 | 6.5 | 113.4 | 45.6 | 18.0 | 17.0 | 11.0 | 22.3 | 153.5 | 134.9 | 72.8 | 207.7 |
| N3P1 | 6.1 | 118.3 | 44.1 | 18.4 | 16.5 | 11.3 | 20.1 | 162.8 | 136.4 | 95.1 | 231.5 |
| N3P2 | 8.6 | 131.1 | 42.5 | 19.8 | 17.5 | 12.6 | 27.5 | 195.1 | 152.0 | 104.2 | 256.1 |
| N3P3 | 7.4 | 125.6 | 43.9 | 19.7 | 18.7 | 11.5 | 24.5 | 186.0 | 168.3 | 99.2 | 267.5 |

**Table S3**. Correlation matrix loadings and variance of the significant principal components (PCs) for grain yield of maize and nitrogen use efficiency (NUE) indices (n = 26)

| Independent variable | Principal components | | |
|---|---|---|---|
| | **PC1** | **PC2** | **PC3** |
| GY | 0.66 | -0.39 | 0.58 |
| SDM | **0.88** | -0.35 | -0.13 |
| TN | **0.84** | -0.54 | 0.06 |
| NHI | -0.68 | 0.26 | 0.62 |
| PFP | 0.35 | **0.90** | -0.15 |
| AN | 0.58 | 0.48 | 0.53 |
| PNB | 0.46 | **0.86** | -0.12 |
| RE | **0.80** | 0.50 | 0.12 |
| IE | **-0.77** | 0.54 | 0.26 |
| PE | 0.08 | 0.10 | **0.73** |
| Ngap | -0.22 | -0.92 | 0.25 |
| Eigenvalue | 4.3 | 3.8 | 1.7 |
| Variance. % | 39.4 | 34.6 | 15.9 |
| Cumulative. % | 39.4 | 74.0 | 89.9 |

Boldfaced loadings are > 0.70. Key: GY – grain yield; SDM – crop residues (straw) biomass; TN – total N accumulation; NHI – N harvest index; PFP - partial factor productivity of N; AE - agronomic efficiency of N; PNB - partial N balance; RE – apparent N recovery efficiency; IE – internal N utilization efficiency; $N_{gap}$ – N gap.

**Table S4**. Pearson correlation matrix between maize grain yield (GY), crop residues dry matter (SDM), total nitrogen accumulation (TN) and nitrogen use indices (n = 26)

| Traits | GY | SDM | TN | NHI | PFP | AN | PNB | RE | IE | PE |
|---|---|---|---|---|---|---|---|---|---|---|
| SDM | 0.63** | | | | | | | | | |
| TN | 0.80*** | 0.90*** | | | | | | | | |
| NHI | -0.16 | -0.74*** | -0.65* | | | | | | | |
| PFP | -0.22 | 0.05 | -0.19 | -0.10 | | | | | | |
| AN | 0.56** | 0.24 | 0.25 | 0.09 | 0.51* | | | | | |
| PNB | -0.12 | 0.15 | -0.07 | -0.15 | 0.99*** | 0.57** | | | | |
| RE | 0.39 | 0.46* | 0.41* | -0.34 | 0.69*** | 0.82*** | 0.76*** | | | |
| IE | -0.52** | -0.89*** | -0.92*** | 0.81*** | 0.16 | -0.00 | 0.05 | -0.32 | | |
| PE | 0.30 | 0.00 | 0.06 | 0.31 | 0.06 | 0.25 | 0.09 | 0.14 | 0.11 | |
| Ngap | 0.35 | 0.07 | 0.32 | 0.09 | -0.95*** | -0.42* | -0.92*** | -0.59** | -0.26 | 0.04 |

***, **, * significant at $p < 0.001$; $p < 0.01$; $p < 0.05$, respectively. Key: GY – grain yield; SDM – crop residues (straw) biomass; TN – total N accumulation; NHI – N harvest index; PFP - partial factor productivity of N; AE - agronomic efficiency of N; PNB - partial N balance; RE – apparent N recovery efficiency; IE – internal N utilization efficiency; $N_{gap}$ – N gap.

**Table S5.** Dry matter and nitrogen management indices depending on fertilization treatment (mean for two years).

| Treatment | Dry matter management indices | | | | Nitrogen management indices | | | |
|---|---|---|---|---|---|---|---|---|
| | DMA | DMR | DMRE | CDMR | NA | NR | NRE | CNR |
| N0P0 | 102.5 ±15.1 | -10.3 ±10.4 | -11.9 ±12.4 | -8.2 ±10.5 | 0.78 ±0.14 | 0.53 ±0.08 | 36.5 ±5.2 | 43.3 ±8.6 |
| N1P0 | 112.2 ±25.0 | -14.1 ±18.6 | -16.5 ±19.9 | -5.7 ±17.9 | 0.79 ±0.32 | 0.74 ±0.21 | 36.8 ±8.1 | 57.9 ±22,3 |
| N1P1 | 106.6 ±22.8 | -8.1 ±19.2 | -24.2 ±25.9 | -4.3 ±18.6 | 0.76 ±0.28 | 0.86 ±0.22 | 38.0 ±10.0 | 57.3 ±14,0 |
| N1P2 | 134.9 ±18.8 | -21.8 ±14.9 | -25.9 ±17.0 | -16.4 ±12.6 | 0.91 ±0.18 | 1.00 ±0.14 | 44.6 ±4.5 | 53.7 ±7,6 |
| N1P3 | 142.1 ±21.2 | -23.2 ±16.9 | -27.1 ±17.3 | -16.6 ±13.3 | 1.40 ±0.26 | 0.67 ±0.17 | 29.0 ±6.9 | 35.4 ±9,2 |
| N2P0 | 120.9 ±16.4 | -15.8 ±13.1 | -21.8 ±15.1 | -13.4 ±13.3 | 1.02 ±0.19 | 0.75 ±0.15 | 33.5 ±5.8 | 44.0 ±9,5 |
| N2P1 | 127.0 ±18.2 | -17.8 ±15.6 | -23.3 ±17.7 | -14.6 ±12.7 | 0.74 ±0.22 | 1.11 ±0.16 | 45.4 ±5.8 | 62.6 ±10,0 |
| N2P2 | 132.4 ±21.4 | -23.0 ±15.9 | -31.2 ±19.8 | -19.0 ±12.7 | 1.07 ±0.35 | 0.86 ±0.22 | 33.2 ±8.5 | 52.3 ±14,8 |
| N2P3 | 151.5 ±18.9 | -28.5 ±15.2 | -36.7 ±18.7 | -21.4 ±11.9 | 1.49 ±0.18 | 0.57 ±0.16 | 24.2 ±5.9 | 27.8 ±7,3 |
| N3P0 | 119.0 ±22.6 | -6.8 ±16.7 | -10.2 ±17.9 | -1.2 ±13.9 | 1.06 ±0.28 | 0.91 ±0.17 | 41.4 ±7.7 | 50.6 ±10,6 |
| N3P1 | 145.2 ±16.9 | -28.9 ±16.0 | -38.8 ±20.9 | -22.7 ±12.5 | 1.31 ±0.25 | 0.74 ±0.16 | 32.8 ±6.6 | 38.9 ±8,8 |
| N3P2 | 133.3 ±19.3 | -13.5 ±17.2 | -22.9 ±17.6 | -10.0 ±15.4 | 1.28 ±0.26 | 0.85 ±0.21 | 33.7 ±7.6 | 41.6 ±11,7 |
| N3P3 | 131.5 ±15.4 | -16.1 ±12.5 | -22.4 ±15.8 | -13.8 ±10.7 | 1.15 ±0.25 | 0.99 ±0.17 | 37.4 ±6.1 | 48.3 ±9,8 |

DMI – dry matter increase ; DMR – dry matter remobilization; NI – nitrogen increase; NR – nitrogen remobilization; DMRE – dry matter remobilization efficiency; CDMR – contribution of DMR assimilates to grain; NRE – N remobilization efficiency; CNR – contribution of remobilized N to grain N content.

**Table S6**. Correlation matrix loadings and variance of the significant principal components (PCs) for grain yield of maize and dry matter and nitrogen management indices (n = 26)

| Independent variable | Principal components | | |
|---|---|---|---|
| | PC1 | PC2 | PC3 |
| GY | 0.68 | 0.57 | -0.32 |
| SDM | **0.93** | 0.11 | 0.08 |
| TN | **0.90** | 0.37 | -0.09 |
| DM2 | -0.56 | **0.76** | -0.13 |
| N*c*2 | **0.91** | -0.02 | 0.36 |
| N*a*2 | 0.58 | **0.71** | 0.34 |
| DMRE | **-0.94** | 0.27 | -0.10 |
| CDMR | **-0.94** | 0.29 | -0.07 |
| NRE | **-0.81** | 0.38 | 0.33 |
| CNR | -0.63 | 0.34 | 0.69 |
| DM1 | **0.92** | 0.11 | 0.10 |
| N*c*1 | -0.18 | **0.77** | -0.32 |
| N*a*1 | **0.88** | 0.32 | 0.01 |
| Eigenvalue | 8.1 | 2.7 | 1.1 |
| Variance. % | 62.3 | 20.6 | 8.4 |
| Cumulative. % | 62.3 | 82.9 | 91.3 |

Boldfaced loadings are > 0.70. Key: GY – grain yield; SDM – crop residues (straw) biomass; TN – total N accumulation; DM1 – dry matter of plant at 7 leaf growth stage; N*c*1 – N concentration at 7 leaf growth stage; N*a*1 - N accumulation at 7 leaf growth stage; DM2 – dry matter of plant at flowering; N*c*2 – N concentration at flowering; N*a*1 - N accumulation at flowering; DMRE – dry matter remobilization efficiency; CDMR – contribution of DMR assimilates to grain; NRE – N remobilization efficiency; CNR – contribution of remobilized N to grain N content.

**Table S7**. Pearson correlation matrix between maize grain yield (GY), crop residues dry matter (SDM), total nitrogen accumulation (TN) and nitrogen content at early growth stages and N remobilization and contribution to grain (n = 26)

| **Traits** | **GY** | **SDM** | **TN** | **DM1** | **N*c*1** | **N*a*1** | **DM2** | **N*c*2** | **N*a*2** | **DMRE** | **CDMR** | **NRE** |
|---|---|---|---|---|---|---|---|---|---|---|---|---|
| SDM | 0.68*** | | | | | | | | | | | |
| TN | 0.85*** | 0.90*** | | | | | | | | | | |
| DM1 | 0.66*** | 0.81*** | 0.83*** | | | | | | | | | |
| N*c*1 | 0.32 | -0.13 | 0.12 | -0.16 | | | | | | | | |
| N*a*1 | 0.75*** | 0.78*** | 0.87*** | 0.96*** | 0.11 | | | | | | | |
| DM2 | 0.10 | -0.40* | -0.19 | -0.44* | 0.61** | -0.29 | | | | | | |
| N*c*2 | 0.47* | 0.84*** | 0.78*** | 0.84*** | -0.22 | 0.79*** | -0.62** | | | | | |
| N*a*2 | 0.68*** | 0.65*** | 0.78*** | 0.61** | 0.31 | 0.69*** | 0.20 | 0.64*** | | | | |
| DMRE | -0.45* | -0.88*** | -0.73*** | -0.82*** | 0.36 | -0.72*** | 0.76*** | -0.90*** | -0.38 | | | |
| CDMR | -0.46* | -0.89*** | -0.73*** | -0.80*** | 0.38 | -0.70*** | 0.77*** | -0.89*** | -0.36 | 0.99*** | | |
| NRE | -0.38 | -0.71*** | -0.65*** | -0.64*** | 0.32 | -0.55** | 0.67*** | -0.63** | -0.12 | 0.83*** | 0.84*** | |
| CNR | -0.46* | -0.47* | -0.51** | -0.49* | 0.18 | -0.44* | 0.51* | -0.33 | 0.10 | 0.60** | 0.63** | 0.85*** |

***, **, * significant at $p < 0.001$; $p < 0.01$; $p < 0.05$, respectively. Key: GY – grain yield; SDM – crop residues (straw) biomass; TN – total N accumulation; DM1 – dry matter of plant at 7 leaf growth stage; Nc1 – N concentration at 7 leaf growth stage; Na1 - N accumulation at 7 leaf growth stage; DM2 – dry matter of plant at flowering; Nc2 – N concentration at flowering; Na1 - N accumulation at flowering; DMRE – dry matter remobilization efficiency; CDMR – contribution of DMR assimilates to grain; NRE – N remobilization efficiency; CNR – contribution of remobilized N to grain N content.
